# Loss of NEIL3 activates radiotherapy resistance in the progression of prostate cancer

**DOI:** 10.20892/j.issn.2095-3941.2020.0550

**Published:** 2021-10-01

**Authors:** Qiong Wang, Zean Li, Jin Yang, Shirong Peng, Qianghua Zhou, Kai Yao, Wenli Cai, Zhongqiu Xie, Fujun Qin, Hui Li, Xu Chen, Kaiwen Li, Hai Huang

**Affiliations:** 1 Department of Urology, Sun Yat-sen Memorial Hospital, Sun Yat-sen University, Guangzhou 510120, China; 2 Department of Pathology, School of Medicine, University of Virginia, Charlottesville, VA 22908, USA; 3 Guangdong Provincial Key Laboratory of Malignant Tumor Epigenetics and Gene Regulation, Sun Yat-sen Memorial Hospital, Sun Yat-sen University, Guangzhou 510120, China; 4 Department of Radiation Oncology, Sun Yat-sen Memorial Hospital, Sun Yat-sen University, Guangzhou 510120, China; 5 Department of Urology, Sun Yat-sen University Cancer Center, Guangzhou 510060, China; 6 Department of Radiology, Massachusetts General Hospital, Harvard Medical School, Boston, MA 02114, USA; 7 Department of Urology, The Sixth Affiliated Hospital of Guangzhou Medical University, Qingyuan People’s Hospital, Qingyuan 511518, China

**Keywords:** CRPC, NEIL3, NEPC, prostate cancer, radiotherapy resistance

## Abstract

**Objective:**

To explore the genetic changes in the progression of castration-resistant prostate cancer (CRPC) and neuroendocrine prostate cancer (NEPC) and the reason why these cancers resist existing therapies.

**Methods:**

We employed our CRPC cell line microarray and other CRPC or NEPC datasets to screen the target gene NEIL3. Lentiviral transfection and RNA interference were used to construct overexpression and knockdown cell lines. Cell and animal models of radiotherapy were established by using a medical electron linear accelerator. Flow cytometry was used to detect apoptosis or cell cycle progression. Western blot and qPCR were used to detect changes in the protein and RNA levels.

**Results:**

TCGA and clinical patient datasets indicated that NEIL3 was downregulated in CRPC and NEPC cell lines, and NEIL3 was correlated with a high Gleason score but a good prognosis. Further functional studies demonstrated that NEIL3 had no effect on the proliferation and migration of PCa cells. However, cell and animal radiotherapy models revealed that NEIL3 could facilitate the radiotherapy sensitivity of PCa cells, while loss of NEIL3 activated radiotherapy resistance. Mechanistically, we found that NEIL3 negatively regulated the expression of ATR, and higher NEIL3 expression repressed the ATR/CHK1 pathway, thus regulating the cell cycle.

**Conclusions:**

We demonstrated that NEIL3 may serve as a diagnostic or therapeutic target for therapy-resistant patients.

## Introduction

Acquired resistance to existing therapies in cancer is an increasing clinical problem. Therapeutic agents exert natural selection and promote the development of therapeutically resistant tumors. Androgen deprivation therapy (ADT) has long been used as a first-line treatment for prostate cancer (PCa) and has proven effective for its early stage^1^, but the development of castration-resistant PCa (CRPC) is nearly inevitable within 2–3 years of initiation of ADT^2^. CRPC is an incurable and rapidly progressing disease state, which mostly is due to the reactivation of androgen receptor (AR) signaling^3,4^. Studies have demonstrated that the median survival of CRPC patients is less than 3 years^5,6^. Although the second-generation hormonal therapy drugs enzalutamide and abiraterone were approved by the Food and Drug Administration (FDA) for CRPC treatment, only some patients responded to the new therapies^7,8^. Unfortunately, more than 25% of CRPC patients will evolve into a more aggressive and treatment-resistant form of neuroendocrine PCa (NEPC)^9^. NEPC is a type of prostate small cell carcinoma that does not express AR or secrete prostate-specific antigen (PSA) but expresses the neuroendocrine markers chromogranin A, synaptophysin (SYP), and neuron-specific enolase (NSE)^10^. Importantly, NEPC always has a very poor prognosis and a probability of survival of less than 1 year^11^. Compared to primary PCa, CRPC and NEPC are both more resistant to existing chemotherapy and radiotherapy^12–14^. CRPC and NEPC are the main causes of prostate cancer-associated mortality, and there are no established therapeutic approaches for their treatment. Therefore, there is an urgent need to identify the regulatory mechanism of CRPC and NEPC occurrence, and clarify the reason why they resist existing therapies.

In this study, we first established 2 LNCaP castration-resistant sublines, named LNcap-AI and LNcap-Bic as in our previous study^15^. Combined with other reported CRPC and NEPC datasets, we compared the changes in gene expression and finally obtained our target gene, Nei endonuclease VIII-like 3 (NEIL3). NEIL3 belongs to a class of the DNA glycosylase family that initiates the first step in base excision repair *via* the associated lyase reaction^16^. In contrast to the other 2 family members NEIL1 and NEIL2, NEIL3 has a more complicated function, including unhooking interstrand cross-links^17,18^, and modulation of DNA methylation^19^. Importantly, studies have shown NEIL3 alterations in PCa^20,21^, but the molecular mechanisms are far from being clarified. Moreover, there is currently no evidence to indicate whether NEIL3 plays a role in the occurrence of CRPC and NEPC, as well as its function in the therapeutic resistance. Here, we found that NEIL3 had a positive correlation with the Gleason score of PCa, but the opposite was true for NEIL3, which indicated a good prognosis. Considering that all our subjects received clinical treatment, we doubt whether NEIL3 was related to treatment sensitivity. Further functional studies implied that loss of NEIL3 activated radiotherapy resistance *via* the ATR/CHK1 pathway by blocking the cell cycle. Here, we demonstrated a possible diagnostic or therapeutic target for clinical therapy-resistant patients.

## Materials and methods

### Data mining

**Figure 1:**
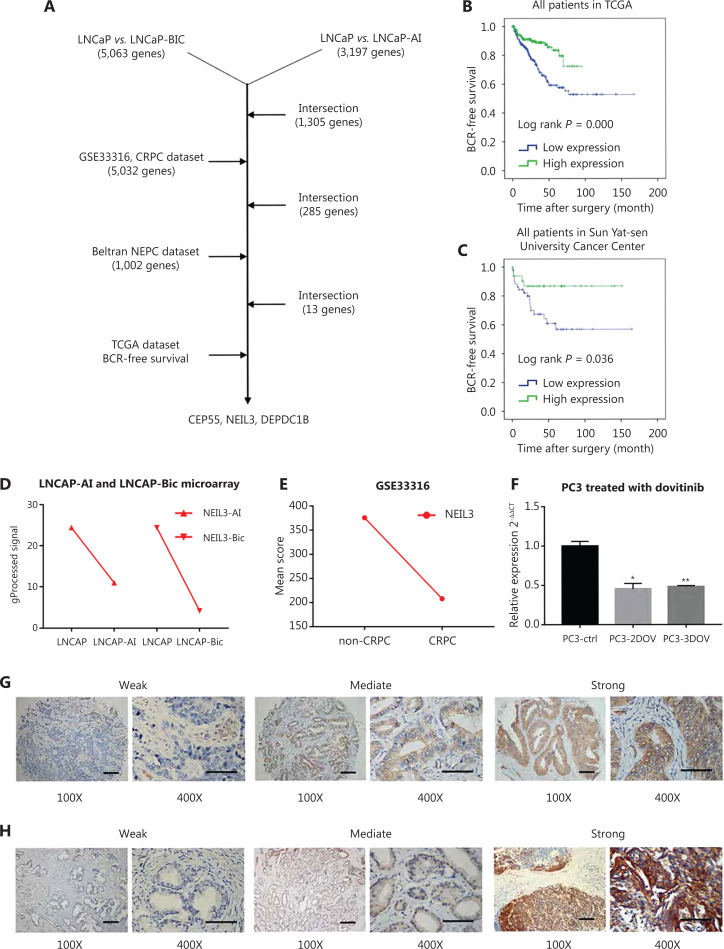
RNA microarray and database-integrated screening identified that NEIL3 correlated with the progression of PCa. (A) Three target genes were selected from the microarray, CRPC database, NEPC database and TCGA BCR survival analysis. (B, C) BCR survival analysis of NEIL3 in TCGA datasets and Sun Yat-sen University Cancer Center patients. (D, E) The expression of NEIL3 in our CRPC microarray and GSE33316. (F) RT-PCR detection of NEIL3 mRNA levels in PC3-derived NEPC cells; *P < 0.05 and **P < 0.01, versus the PC3-ctrl group. (G, H) Representative active immunohistochemical staining and quantification showing the expression level of NEIL3 in TMA (G) and Sun Yat-sen University Cancer Center (H) PCa tissues.

For screening target genes, we downloaded a CRPC dataset from the Gene Expression Omnibus (https://www.ncbi.nlm.nih.gov/geo/query/acc.cgi): GSE33316. Additionally, we downloaded another NEPC dataset from Beltran’s research^22^. To further select target genes by biochemical recurrence (BCR)-free survival rate, we downloaded the patient clinical profiles from The Cancer Genome Atlas (TCGA) prostate adenocarcinoma datasets. Then, we used SPSS 20.0 software to depict the BCR-free survival rate and to obtain our target gene. The screening process is shown in **Figure 1A**.

TCGA databases were used to explore the differential NEIL3 expression at the mRNA level in different patients. All 497 patients were used for the analysis as were the 78 patients who had received radiotherapy. The relationship between NEIL3 level and clinicopathological features of the patients are listed in **Supplementary Table S1** (all 497 patients) and **Table 1** (radiotherapy, 78 patients).

**Table 1:** Correlation of NEIL3 expression with clinico-pathologic characteristics of PCa patients in TCGA database (78 radiotherapy patients)

Clinical features	Total patients, *n*	Low, *n* (%)	High, *n* (%)	*P*
Age, years			0.194
≤ 65	58	24 (41.4%)	34 (58.6%)
> 65	20	5 (25%)	15 (75%)
Gleason score			0.017*
≤ 7	18	11 (61.1%)	7 (38.9%)
> 7	60	18 (30%)	42 (70%)
Serum PSA levels, ng/mL			0.826
≤ 4	66	25 (37.9%)	41 (62.1%)
> 4	6	2 (33.3%)	4 (66.7%)
T stage			0.051
T1-T2	8	6 (75%)	2 (25%)
T3-T4	70	23 (32.9%)	47 (67.1%)
Lymph node metastasis			0.492
N0	44	15 (34.1%)	29 (65.9%)
N1	26	11 (42.3%)	15 (57.7%)
Distant metastasis			1.000
M0	70	26 (37.1%)	44 (62.9%)
M1	1	0 (0%)	1 (100%)

Cut off value of NEIL3 score: medium; PSA, prostate‑specific antigen; T, tumor; N, node; M, metastasis. *P < 0.05.

### Patients and tissue samples

A tissue microarray (TMA; *n* = 192), including 160 PCa tissue samples and 16 adjacent or normal prostate tissue samples, was purchased from Xi’an Alena Biotechnology Ltd., Beijing, China (catalog no. PR1921c), and the detailed clinical information was included.

A total of 99 paraffin-embedded PCa tissues were obtained from Sun Yat-sen University Cancer Center (Guangzhou, China) from January 2000 to August 2018. The uses of tissues were approved by Sun Yat-sen University’s Committees for Ethical Review of Research Involving Human Subjects (Approval No. SYSEC-KY-KS-2020-201). All samples were diagnosed with PCa by 2 independent clinicians in the Department of Pathology. The clinical information, including TNM stage, Gleason score, PSA levels, T stage, lymph node metastasis and distant metastasis, was obtained according to the guidelines with written consent from the patients.

### Immunohistochemistry staining and scoring analyses

Ki67 (1:500; Servicebio; Wuhan, China) and NEIL3 antibodies (1:300; ab230908; Abcam, Cambridge, UK) were used to assess the protein level in PCa tissues and TMA were used for mouse tumors *via* immunohistochemistry (IHC) according to standard procedures. The images were acquired for statistical analysis using a Nikon Eclipse 80i system (Nikon, Tokyo, Japan). NEIL3 protein expression in the PCa samples was blindly quantified by 2 researchers. First, we evaluated the immunostaining intensity of each sample as follows: negative = 0, weak = 1, moderate = 2, and strong = 3. Second, we assessed the proportion of positively stained cells: < 25% = 1, 25%–50% = 2, 51%–75% = 3 and > 75% = 4. The immunoreactivity score (IRS) was calculated as the intensity score multiplied by the proportion score. The results were divided into 2 groups: low (IRS ≤ 6) and high (IRS > 6).

### The construction of CRPC and NEPC stable cells

Human PCa-related cell lines (PC3 and DU145) and the kidney cell line, 293T, were obtained from American Type Culture Collection (ATCC, Manassas, VA, USA). All cell culture conditions were performed according to the guidelines from ATCC. The culture medium (such as DMEM and RPMI 1640), fetal bovine serum (FBS) and penicillin/streptomycin were all purchased from Gibco, Shanghai, China. All cells were cultured at 37 °C with 5% humidified CO_2_ (BB150, Thermo Scientific, Beijing, China).

We constructed 2 CRPC cell sublines (LNCaP-Bic and LNCaP-AI) as described in our previous study^15^. In detail, for LNCaP-Bic cells, bicalutamide was added to the culture medium at a starting concentration of 5 mM, with a weekly increment of 50% concentration, ultimately being maintained at 20 mM. For LNCaP-AI, LNCaP cells were cultured in medium containing 10% charcoal-stripped FBS (cs-FBS). Both sublines were cultured for 12 months to obtain stable cells.

We constructed NEPC cell sublines as described in a previous study^23^. In detail, PC3 cells were treated with medium containing dovitinib (SelleckChem, Houston, USA) at concentrations of 2 µM and 3 µM until they reached 70%–80% confluency. Medium with dovitinib was refreshed every 3 days. Both sublines (PC3-2DOV and PC3-3DOV) were cultured for at least 1 month to obtain stable cells.

### Transient transfection and plasmid construction

RNA interference (siRNA) oligonucleotides targeting NEIL3 and negative control siRNAs were purchased from GenePharma (Shanghai, China). The siRNA sequences are listed in **Supplementary Figure S1A**. Transient siRNA transfection was carried out as described in a previous study^24^. The NEIL3 sequence was cloned into the pLV-CMV-MCS-EF1-ZsGreen1-T2A-puro vector (Fenghui Biotechnology, Hunan, China) to construct the overexpression plasmid (**Supplementary Figure S1B**). Acquisition of lentivirus, packaging of lentivirus, and screening of stable cells were carried out as described in our previous study^24^.

### Cell proliferation and migration assay

The MTS assay and colony formation assay were used to test cell proliferation. Cells (1,000 for DU145 and 1,500 for PC3 cells per well) were seeded in 96-well plates and cultured for 3 days. We detected the absorbance of each well at 492 nm every day using MTS (Promega, Beijing, China). In detail, 20 µL MTS was added to each well, and then the 96-well plate was incubated at 37 °C for 2 h incubation. The same cells were seeded in 6-well plates and cultured for 14 (control) or 21 (radiation) days for colony formation. Then, colonies were fixed with 4% paraformaldehyde, stained with 0.2% Crystal Violet, and counted.

A 24-well Transwell chamber (8 µM, 353097; Corning, Glendale, USA) was used for the migration assay. In detail, 40,000 cells in 200 µL of 1% FBS medium were seeded in the top chamber, and 600 µL of medium containing 10% FBS was added into the lower chamber. The time of Transwell assay was 10 h (DU145) and 48 h (PC3) for migration. Then, the chamber was fixed with 4% paraformaldehyde and stained with 0.2% Crystal Violet. A microscope (Nikon, Tokyo, Japan) was used to detect the number of migrated cells on the lower membrane surface of the top chamber.

All the experiments above were performed 3 times.

### Cell radiation exposure

Transfected cells were exposed to 2–8 Gy irradiation using a medical electron linear accelerator (PRIMUS, Baden-Württemberg, Germany) with a fixed emission dose rate of 200 MU/min. The source cell distance was 10 cm, and the field size was 25 cm × 25 cm.

### RNA isolation and qRT-PCR

Total RNA was isolated using RNAiso Plus (TaKaRa Bio, Shiga, Japan) according to standard procedures. The PrimeScript RT Reagent Kit (RR047A; TaKaRa) was used to synthesize cDNA. Quantitative real-time PCR was carried out with TB Green Premix Ex TaqII (TaKaRa) in an ABI QuantStudio Sequence Detection System (Applied Biosystems, Foster City, CA, USA). **Supplementary Table S3** lists the sequences of the primers.

### Protein isolation and Western blot

The proteins in the cell samples were harvested using RIPA lysis buffer (Beyotime, Nanjing, China) and separated by 10% sodium dodecyl sulfate polyacrylamide gel electrophoresis. Then, the proteins were transferred to polyvinylidene fluoride membranes and incubated with the following primary antibodies at 4 °C for 16 h: NEIL3 (diluted 1:1,000; ab230908; Abcam), GAPDH [diluted 1:1,000; 97166S; Cell Signaling Technology (CST), Danvers, MA, USA], TOPBP1 (diluted 1:1,000; 14342S; CST), pATR (diluted 1:1,000; 2853S; CST), ATR (diluted 1:1,000; 2790S; CST), pCHK1 (diluted 1:1,000; 2344S; CST), and γ-H2AX (diluted 1:1,000; 7631S; CST). The membranes were then incubated with secondary antibody at room temperature for 1 h. The protein band signals were detected by Immobilon Western Chemiluminescent HRP Substrate (WBKLS0500, Merck Millipore, Darmstadt, Germany).

### Flow cytometry

Flow cytometry (Beckman CytoFLEX, San Jose, CA, USA) was used to detect apoptosis or the cell cycle. For apoptosis, 5 × 10^5^ cells were washed twice with PBS and resuspended in 100 µL 1 × binding buffer. The cells with green fluorescence were mixed with a 10 µL Annexin V-APC/7-AAD apoptosis kit reagent (abs50008; Absin, Shanghai, China), while the cells without green fluorescence were mixed with a 10 µL Annexin V-FITC/PI apoptosis kit reagent (E-CK-A211, Elabscience, Wuhan, China). Then, the cell suspension was incubated at room temperature for 15 min and resuspended in 300 µL binding buffer for flow cytometry detection. Regarding the cell cycle, 5 × 10^5^ harvested cells were washed twice with PBS and then fixed in 70% prechilled ethanol at 4 °C overnight. Cells were then resuspended in 50 µL RNase and 300 µL propidium iodide (PI) buffer and incubated at room temperature for 30 min for flow cytometry detection. ModFit software (BD) was used to analyze the results.

### Xenografts and radiotherapy in mice

Male BALB/c nude mice (4 weeks old) were purchased from the Experimental Animal Center of Sun Yat-sen University and housed in the Laboratory Animal Center of Sun Yat-sen University. All animal procedures were approved and supervised by the Animal Ethics Committee of Sun Yat-sen University (Approval No. SYSU-IACUC-2019-B459). To evaluate the role of NEIL3 in radiotherapy resistance in PCa, a subcutaneous tumorigenic animal model was used in our study. Five million DU145 cells (negative control and stably overexpressed NEIL3) were injected subcutaneously into the left side of the dorsum, and 10 mice were used in each group. Five mice in each group received 5 Gray single-dose irradiation using an X-ray irradiator (Rs 2,000, Rad Source, Buford, GA, USA) when the volume reached approximately 550 mm^3^ (length × width^2^ × 0.5). Another 5 mice in each group were sacrificed for immunohistochemical analyses. The irradiation dose of 5 Gray was determined based on our previous experiments and published studies^25,26^. The volumes of the tumors were calculated every 2 days, and the mice were sacrificed 16 days after radiotherapy with the tumors being surgically dissected. The removed tumors were fixed in 10% buffered formalin for immunohistochemical analyses.

### Statistical analysis

All primary data from TCGA, TAM and clinical samples were analyzed using SPSS 22.0 software (SPSS Inc., Chicago, IL, USA). Pearson’s chi-squared and Fisher’s exact tests were used to analyze the association of NEIL3 expression with clinicopathological characteristics. The Kaplan-Meier method was used to describe BCR-free survival, and *P* < 0.05 was considered significant after the log-rank test.

All quantitative data are presented as the mean ± SD and were evaluated using GraphPad Prism 5.0 (GraphPad, La Jolla, CA, USA). Statistical differences between the groups were assessed by one-way analysis of variance followed by Student’s *t*-test, and *P* < 0.05 was considered significant.

## Results

### RNA microarray and database-integrated screening showed that NEIL3 correlated with the progression of PCa

To study the aberrantly expressed mRNAs in CRPC and NEPC cells, we first established 2 LNCaP castration-resistant sublines that are considered to best simulate the clinical progression of CRPC^27^. Our previous study proved that these 2 LNCaP castration-resistant sublines were resistant to bicalutamide and could proliferate well under androgen deprivation conditions^15^. More importantly, the level of PSA was significantly downregulated, while AR, AR-V7, c-Myc, and bcl-2 were upregulated in LNCaP-Bic and LNCaP-AI cells.

The RNA microarray was used to screen for differentially expressed genes between androgen-dependent and androgen-independent PCa cells. We identified 5,063 differentially expressed genes between LNCaP-Bic and LNCaP cells (3,293 genes with low expression and 1,770 genes with high expression in LNCaP-Bic) and 3,197 differentially expressed genes between LNCaP-AI and LNCaP cells (1,760 genes with low expression and 1,437 genes with high expression in LNCaP-AI) of which there were 1,305 intersections. To narrow further the screening scope, we merged the sequencing results from a CPRC sample (GSE33316, 5,032 genes; 2,271 were low expression, while, 2,761 were highly expressed in the CRPC sample) and a Beltran’s NEPC dataset (1,002 genes; 481 were low expression, while 521 were highly expressed in the NEPC sample)^22^. Finally, 13 candidate genes related to CRPC and NEPC were selected (**Figure 1A and Supplementary Materials**). TCGA databases were used to describe the BCR survival curve of these 13 genes to explore the relationship between the 13 genes and the prognosis of PCa (**Figure 1B–1D, Supplementary Figure S2**). However, only NEIL3, CEP55, and DEPDC1B had a significant relationship with the prognosis of prostate cancer. Furthermore, our data showed that DEPDC1B, CEP55, and NEIL3 were significantly reduced in the CRPC cell line (**Supplementary Figure S2B–S2C, Figure 1D–1E**). In addition, we further confirmed a low level of NEIL3 in the NEPC cell line (**Figure 1F**). Although some studies have found that NEIL3 is mutated in PCa patients, the reason is unknown^20^. In summary, loss of NEIL3 may play an important role in the progression of PCa.

### NEIL3 was correlated with a high Gleason score but a good prognosis

To further investigate whether NEIL3 was involved in clinical PCa progression at the mRNA and protein levels, we analyzed TCGA datasets, consisting of 497 patients, which included clinicopathological characteristics. We found that a high mRNA level of NEIL3 was related to a higher Gleason score (*P* = 0.000) and T stage (*P* = 0.000) and indicated a higher possibility of lymph node metastasis (*P* = 0.006) (**Supplementary Table S1**). Considering that mRNA levels sometimes fail to reflect the true level of a protein, we further analyzed the protein level in a tissue microarray (**Figure 1G and Supplementary Figure S3**) and a large-scale sample cohort containing 99 PCa specimens (**Figure 1H**). Statistical analyses showed that a high protein level of NEIL3 was related to a high Gleason score (*P* = 0.015 and 0.036, respectively) but not to PSA level, T stage, lymph node metastasis, or distant metastasis (**Table 2 and Supplementary Table S2**). Combined with TCGA results, we were confident that NEIL3 was associated with a high Gleason score, which suggested it may be an oncogene. Interestingly, Kaplan-Meier survival analysis showed that high mRNA or protein levels of NEIL3 were correlated with a good prognosis in TCGA and clinical cohorts (*P* = 0.000 and 0.036, respectively) (**Figure 1B and 1C**). Taken together, NEIL3 was correlated with a high Gleason score but a good prognosis, and the reason needs to be further explored.

**Table 2:** Correlation of NEIL3 expression with clinico-pathologic characteristics of PCA patients in Sun Yat-sen University Cancer Center (99 patients)

Clinical features	Total patients, *n*	Low, *n* (%)	High, *n* (%)	*P*
Age, years			1.000
≤ 65	44	28 (63.6%)	16 (36.4%)
> 65	55	35 (63.6%)	20 (36.4%)
Gleason score			0.036*
≤ 7 (3 + 4)	44	33 (75%)	11 (25%)
≥ 7 (4 + 3)	55	30 (54.5%)	25 (45.5%)
Serum PSA levels, ng/mL			0.751
≤ 4	35	23 (65.7%)	12 (34.3%)
> 4	64	40 (62.5%)	24 (37.5%)
T stage			1.000
T1–T2	20	15 (75%)	5 (25%)
T3–T4	36	28 (77.8%)	8 (22.2%)
Lymph node metastasis			0.654
N0	38	29 (76.3%)	9 (23.7%)
N1	28	20 (71.4%)	8 (28.6%)
Distant metastasis			0.586
M0	21	15 (71.4%)	6 (28.6%)
M1	22	14 (63.6%)	8(36.4%)

Cut off value of immuno-reactivity score: 6 (> 6, High; ≤ 6, Low.); T, tumor; N, node; M, metastasis. *P < 0.05.

### NEIL3 had no effect on the proliferation and migration of PCa cells *in vitro*

**Figure 2:**
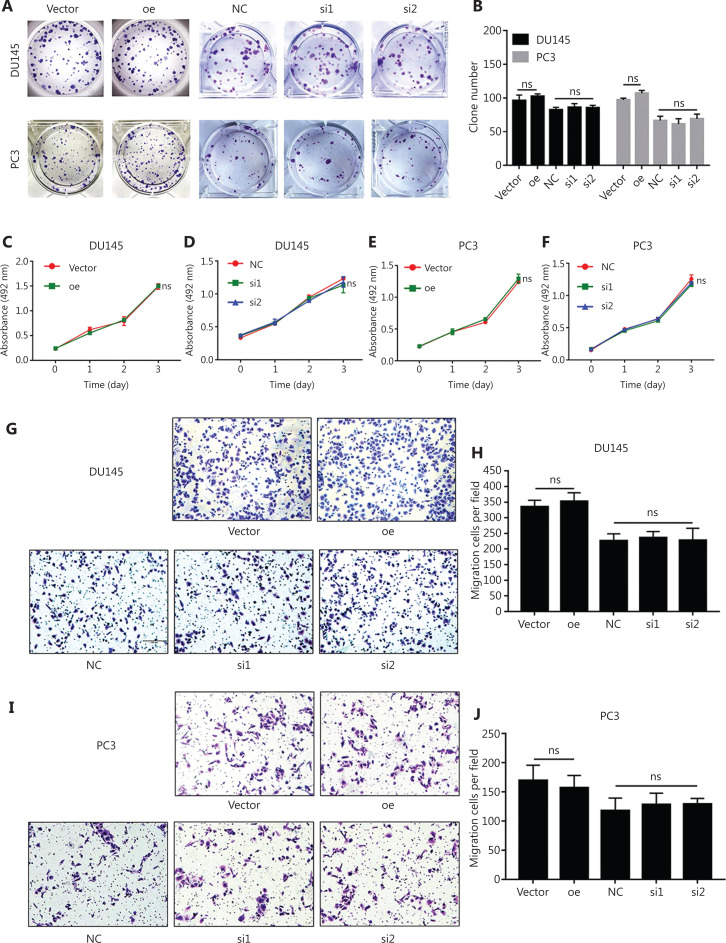
NEIL3 has no effect on the proliferation and migration of PCa cells *in vitro.* (A) Colony formation assay test cell viability in DU145 and PC3 cell lines when NEIL3 was overexpressed or downregulated. (B) Statistical analysis of the number of colonies between different groups. (C–F) The MTS assay test of cell viability in DU145 (C, D) and PC3 (E, F) cells when NEIL3 was overexpressed or downregulated. (G and I) Representative images of migration assays using DU145 and PC3 cells after downregulation or upregulation of NEIL3. (H and J) Histogram analysis of migrated cell counts showing cell migration after downregulation or upregulation of NEIL3.

To explore the effect of NEIL3 on prostate cancer, DU145 and PC3 cell lines were transfected with 2 small interfering RNAs to construct a NEIL3 knockdown cell line (**Supplementary Figure S1A**) or infected with the corresponding packaged lentivirus to establish a stable NEIL3-overexpressing cell line (**Supplementary Figure S1B**). RT-PCR and Western blot showed that the mRNA and protein levels of NEIL3 were downregulated or increased in DU145 and PC3 cell lines (**Supplementary Figure S1C–S1F**). MTS assays and colony formation assays were used to detect NEIL3 function in PCa viability. It was found that whether NEIL3 was overexpressed or knocked down, it had no effect on the proliferation of DU145 and PC3 cells (**Figure 2A–2F**). Similarly, Transwell assays showed that NEIL3 did not influence the migration of DU145 and PC3 cells (**Figure 2G–2J**). Taken together, we believe that NEIL3 improved the prognosis of prostate cancer, but did not directly affect its proliferation or metastasis.

### NEIL3 facilitated radiotherapy sensitivity of PCa cells *in vitro* and *in vivo*

**Figure 3:**
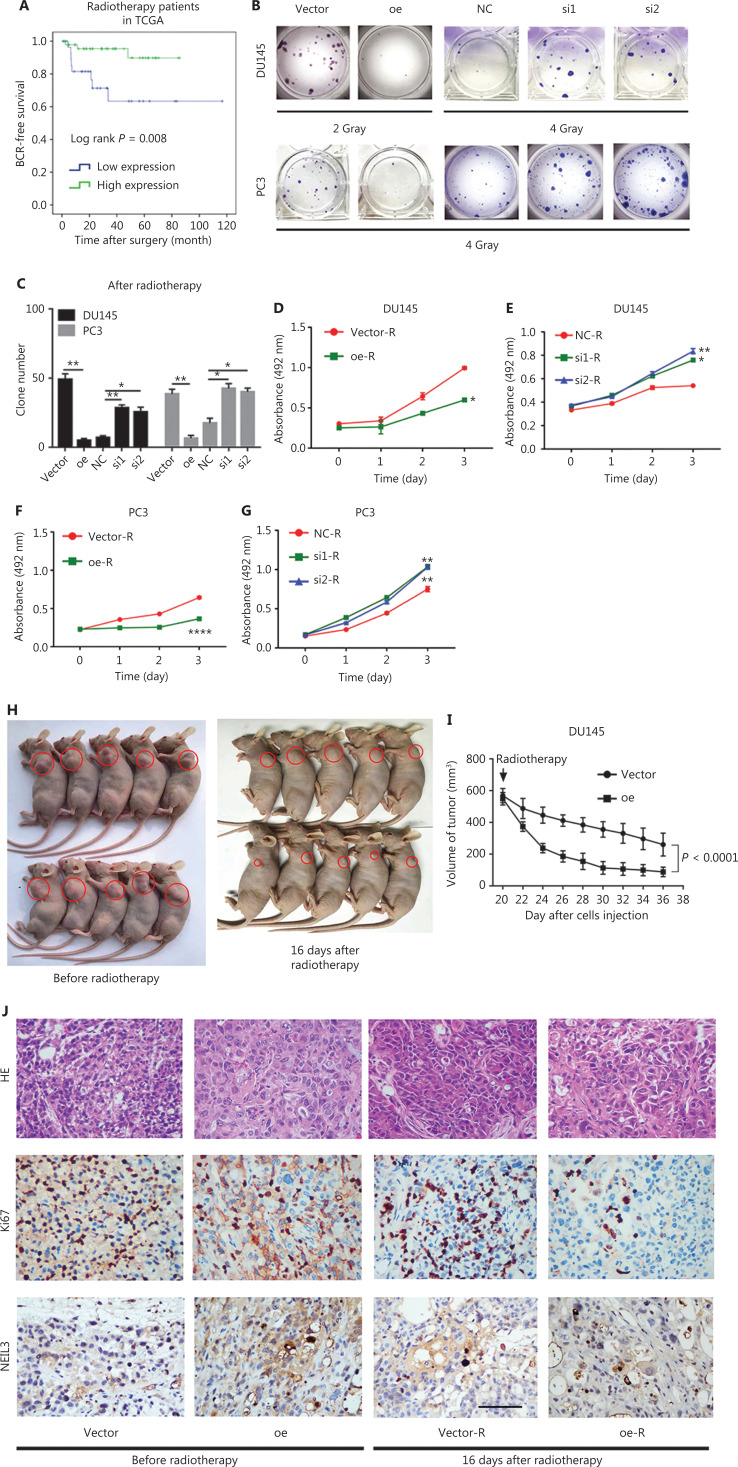
NEIL3 facilitates radiotherapy sensitivity of PCa cells *in vitro* and *in vivo*. (A) BCR survival analysis of NEIL3 in TCGA radiotherapy patients. (B) Colony formation assay test cell viability in DU145 and PC3 cell lines after radiotherapy when NEIL3 was overexpressed or downregulated. (C) Statistical analysis of the number of colonies between different groups; *P < 0.05 and **P < 0.01 between groups. (D–G) The MTS assay test of cell viability in DU145 (D, E) and PC3 (F, G) cells after radiotherapy when NEIL3 was overexpressed or downregulated; *P < 0.05, **P < 0.01 and ****P < 0.0001, versus the corresponding vector or NC group. (H) Gross observation of the nude BALB/c xenografts and radiotherapy mouse model. (I) Tumor reduction curves of the NEIL3 overexpression and control groups are summarized in the line chart after radiotherapy. The average tumor volume is expressed as the mean ± SD. (J) Representative HE and immunohistochemical staining of xenograft tumors before and after radiotherapy; 400×, scale bars = 100 μm.

All our human research subjects had received medical treatment, including endocrine therapy, chemotherapy or radiotherapy. Our depictions of BCR survival rates cannot exclude these treatment factors. In addition, CRPC and NEPC are both more resistant to existing therapies than primary PCa. Therefore, we hypothesized that NEIL3 was related to the treatment sensitivity of PCa patients. To validate this hypothesis, we selected 78 patients in TCGA database who had received radiation therapy, evaluated the correlation of NEIL3 with clinicopathological characteristics, and depicted the BCR survival rates. Similarly, high NEIL3 was related to a higher Gleason score (*P* = 0.017) (**Table 1**), but indicated a good prognosis (*P* = 0.008) (**Figure 3A**). More interestingly, MTS assays and colony formation assays demonstrated that after the cells received 4 Gray or 2 Gray radiation, the overexpression group showed significant promotion of radiation sensitivity, while the corresponding knockdown group showed significant reduction of radiation sensitivity (**Figure 3B–3G**). We chose 4 Gray as the target dose because a higher dose will cause the death of most cells (**Supplementary Figure S4A and S4B**). For the DU145 overexpression group, we chose 2 Gray as the target dose because DU145 was more sensitive to radiotherapy than PC3 cells. Even 4 Gray caused significant damage to DU145 cells (**Supplementary Figure S4C**).

To further explore the effects of NEIL3 on the radiotherapy sensitivity of PCa cells *in vivo*, a xenograft and radiotherapy mouse model was used. DU145 cell lines with stable overexpression of NEIL3 were subcutaneously injected into BALB/c nude mice. During the 20 days of tumor formation, we did not observe a difference in tumor volume between the control and NEIL3-overexpressing groups (**Figure 3H and 3I**). On the 20th day, when the tumor volume reached approximately 550 mm^3^, the tumor site received radiation therapy, and the size of the tumors was measured every 2 days in the next half month. Strikingly, the tumor volume of the NEIL3 upregulation group shrank faster than that of the control group (**Figure 3H and 3I**). Moreover, before radiotherapy, we did not detect a difference in the proliferation marker Ki67 between the control and NEIL3-overexpressing groups, while after radiotherapy, the NEIL3-overexpressing group exhibited much lower expression of Ki67. In addition, we also found that most of the NEIL3-positive cells disappeared after radiotherapy (**Figure 3J**). Taken together, NEIL3 facilitated the radiotherapy sensitivity of PCa *in vitro* and *in vivo*.

### NEIL3 facilitated radiotherapy sensitivity by regulating the cell cycle

**Figure 4:**
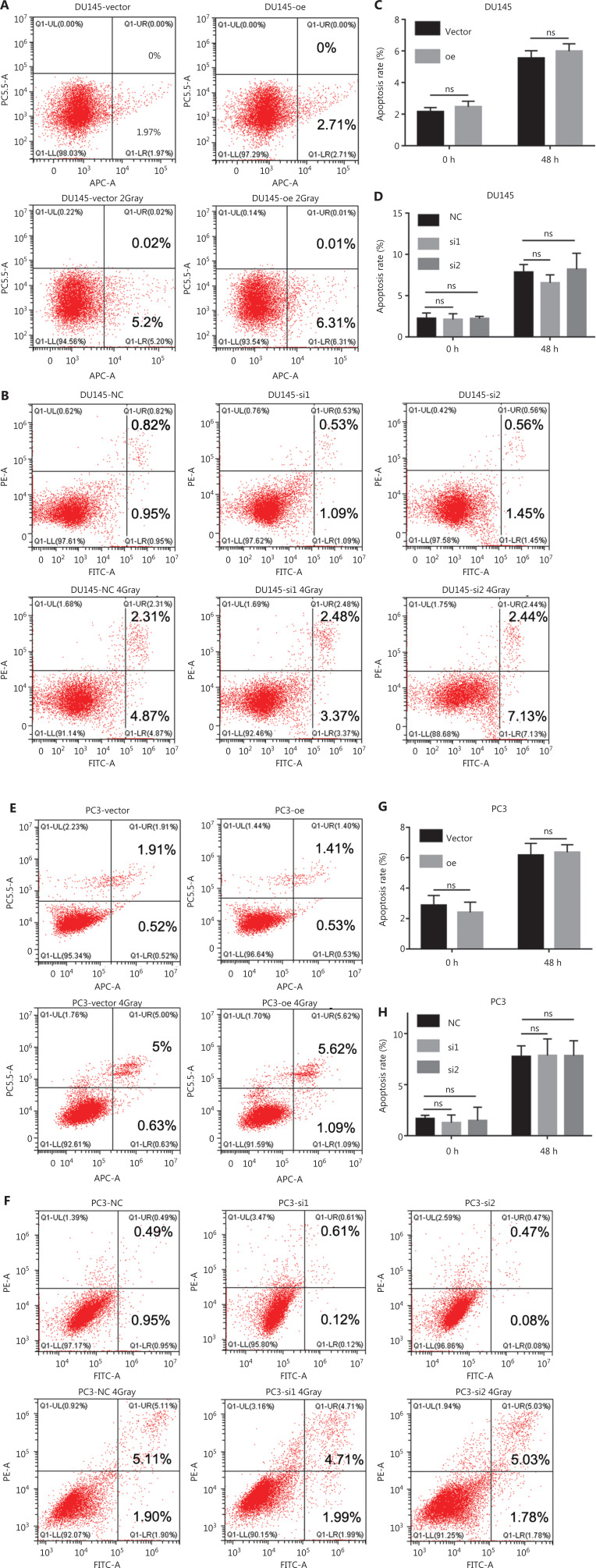
NEIL3 promotes radiotherapy sensitivity by not affecting apoptosis. (A–D) Representative flow cytometry cell apoptosis images (A, B) and histogram analysis of apoptotic cell counts (C, D) of DU145 before and 48 h after radiotherapy in the NEIL3 overexpression or NEIL3 knockdown groups. (E–H) Representative flow cytometry cell apoptosis images (E, F) and histogram analysis of apoptotic cell counts (G, H) of DU145 before and 48 h after radiotherapy in the NEIL3 overexpression or NEIL3 knockdown groups.

**Figure 5:**
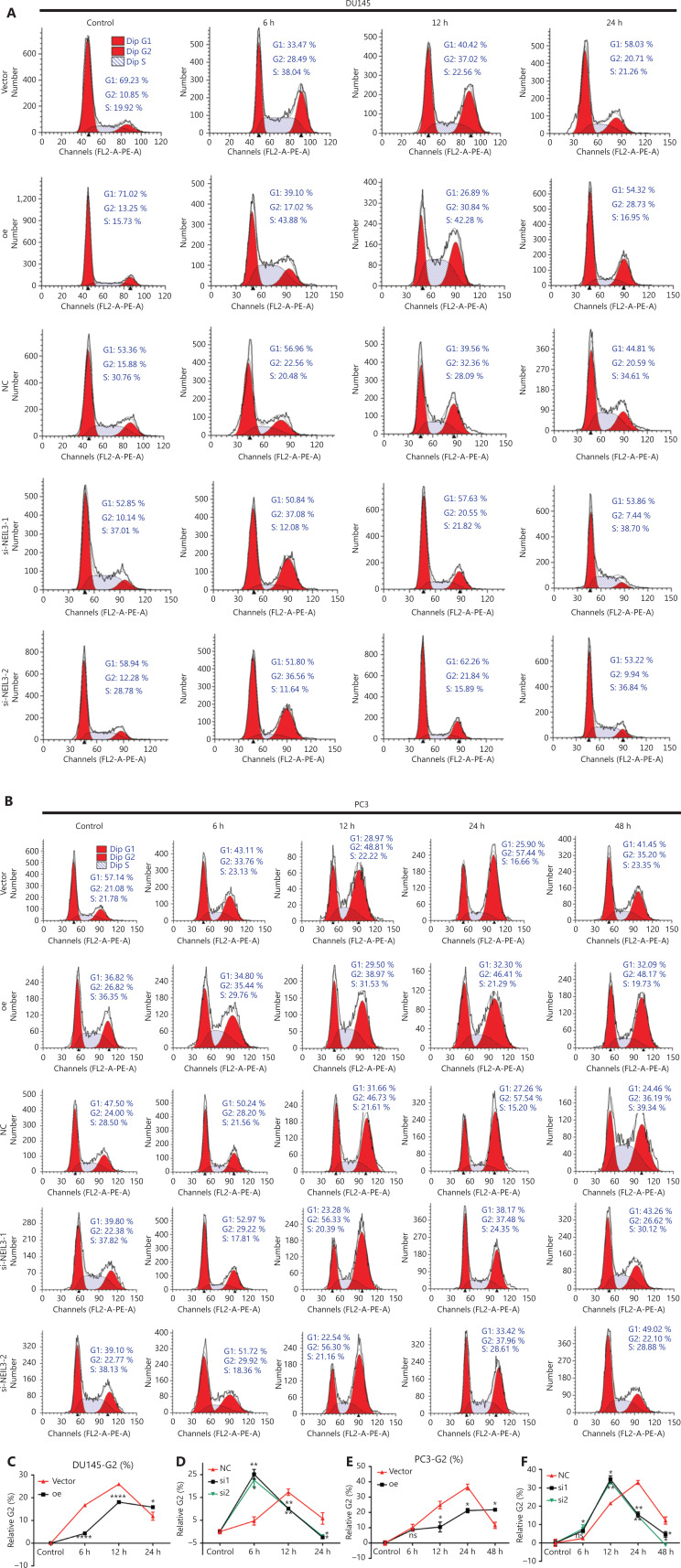
NEIL3 promotes radiotherapy sensitivity by regulating the cell cycle. (A and B) Representative flow cytometry cell cycle images of DU145 (A) and PC3 (B) cells before and 6 h, 12 h, and 24 h after radiotherapy. (C–F) Relative G2-phase change curves of NEIL3 overexpression (C, E) or knockdown (D, F) in DU145 and PC3 cell lines; *P < 0.05, **P < 0.01, and ****P < 0.0001, versus the corresponding time point vector or NC group.

We found that NEIL3 increased the inhibitory effect of radiotherapy on the proliferation of PCa. The level of apoptosis and the blockage of the cell cycle are the 2 important factors affecting cell proliferation. Therefore, through the GEPIA website (http://gepia.cancer-pku.cn/), we found that NEIL3 was positively correlated with the levels of the apoptosis-related genes, BAX and PCNA (**Supplementary Figure S5A–S5C**). Similarly, the level of NEIL3 was also positively correlated with the cell cycle-related genes, TOPBP1, ATR, ATM, CHK1, CHK2, and CDK1 (**Supplementary Figure S5D–S5I**). Therefore, we further performed flow cytometry on PCa cells at 48 h after radiotherapy and found that although radiotherapy caused a certain degree of apoptosis, overexpression or knockdown of NEIL3 had little effect on apoptosis (**Figure 4**). Thus, we suspected that NEIL3 may facilitate radiotherapy sensitivity by regulating the cell cycle. Flow cytometry results indicated that PCa cells underwent G2 arrest after receiving radiation therapy. Moreover, the peak of cell cycle arrest appeared at 12 h in DU145 cells and 24 h in PC3 cells, and cell cycle arrest recovered within 24 h in DU145 cells and 48 h in PC3 cells (**Figure 5A and 5B**). Interestingly, we found that although radiation also blocked the cell cycle in the NEIL3-overexpressing group, its reaction time was slower than that in the control group. More importantly, cell cycle arrest persisted after radiotherapy and was more difficult to recover than that in the control group (**Figure 5C–5E**). In contrast, the occurrence and recovery of cell cycle arrest were both faster in the NEIL3-downregulation group than in the control group (**Figure 5D–5F**). Cell cycle arrest has long been considered a self-protection mechanism that will occur when facing replication stress. Blocking the cell cycle allows cells to obtain enough time to repair DNA to avoid spreading this damage to the next generation, thus protecting against genomic instability. Here, we found that NEIL3 could reduce the responsiveness of PCa cells to replication stress, slow down cell cycle arrest, and thus prevent DNA damage from being repaired. We believe that this accumulation of genomic damage leads to the instability of the genome, which ultimately causes cell death.

### NEIL3 regulated the cell cycle through the ATR/CHK1 pathway

DNA damage checkpoints employ damage sensor proteins, such as ATM and ATR, to detect DNA damage and to inhibit cell cycle progression. There are different DNA damage checkpoints in different phases within the cell cycle. Studies have demonstrated that the activation of the TOPBP1/ATR/CHK1 pathway induces cell cycle arrest at G2 phase^28,29^. To explore the mechanism by which NEIL3 regulates G2-phase arrest, Western blot was used to detect the effect of NEIL3 on the TOPBP1-ATR-CHK1 pathway and DNA damage.

**Figure 6:**
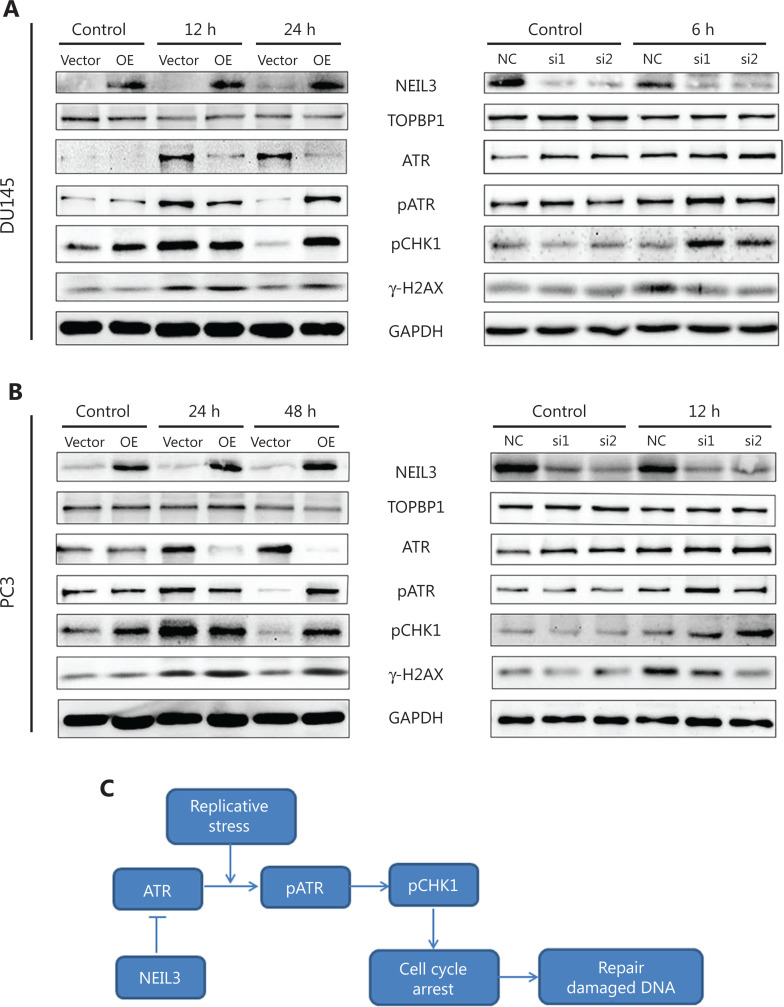
NEIL3 regulates the cell cycle through the ATR/CHK1 pathway. (A and B) Representative image of the Western blot analysis of NEIL3, TOPBP1, ATR, phosphorylated ATR, phosphorylated CHK1 and γ-H2AX protein levels after NEIL3 knockdown or overexpression in DU145 (A) and PC3 (B) cells. (C) Pathway illustration of NEIL3 influencing the ATR/CHK1 pathway.

Before radiotherapy, we found that overexpressed NEIL3 could increase the level of pCHK1 (phosphorylated CHK1), which may be the reason why the overexpressed group had a slightly higher percentage of cells in G2 phase (**Figure 5A–5B**), while knocking down NEIL3 could increase the level of ATR. Considering that ATR is phosphorylated and activated when faced with replication stress, we hypothesized that ATR is expressed at low levels under normal conditions and that NEIL3 may act as a switch for ATR expression. To verify this hypothesis, we extracted the proteins from PCa cells after radiotherapy and performed Western blot. From the change in γ-H2AX, we found that PCa cells showed obvious DNA damage after radiotherapy, and the damage was completely repaired 24 h after radiotherapy in DU145 cells (or 48 h in PC3 cells). Moreover, pATR (phosphorylated ATR) and pCHK1 showed the same trend as γ-H2AX, which meant that the ATR/CHK1 pathway was activated to arrest the cell cycle when DNA damage occurred and the pathway was silenced, and cell cycle re-entry occurred when repair was completed. In addition, the activation of the ATR/CHK1 pathway was much slower in the NEIL3-overexpressing group than in the control group. We were still able to detect activation of the ATR/CHK1 pathway and high levels of γ-H2AX even 24 h (DU145)/48 h (PC3) after radiotherapy when NEIL3 was overexpressed. In contrast, activation of the ATR/CHK1 pathway was much faster in the NEIL3 downregulation group than in the control group. The levels of γ-H2AX were much lower at 6 h (DU145)/12 h (PC3) after radiotherapy when NEIL3 was knocked down. Moreover, we did not observe a significant change in TOPBP1 regardless of how NEIL3 changed (**Figure 6**). Taken together, we hypothesized that NEIL3 may act as a switch for ATR expression: under normal conditions, high NEIL3 inhibits the expression of ATR, and the ATR/CHK1 pathway is silenced, ensuring the proliferation of cells. When faced with replication stress, the decrease in NEIL3 allows the expression of ATR, and the ATR/CHK1 pathway is activated to arrest the cell cycle for DNA repair. If NEIL3 persists at a high level, ATR is suppressed, the ATR/CHK1 pathway cannot be fully activated, and DNA cannot be repaired in time. In contrast, if NEIL3 persists at a low level, ATR is relatively high even under normal conditions, and the ATR/CHK1 pathway could be activated faster when facing replication stress and DNA damage can be rapidly repaired.

## Discussion

CRPC and NEPC are the main causes of death in PCa patients. However, the regulatory mechanism of CRPC and NEPC occurrence and the reason why they resist existing therapies are far from elucidated. Here, based on the established CRPC cell line microarray data and CRPC and NEPC datasets, we screened the target gene, NEIL3, and found its low expression in CRPC and NEPC cell lines. TCGA and our clinical patient cohort indicated that NEIL3 was correlated with a high Gleason score but a good prognosis. Considering that the patients had received medical treatment, we hypothesized that NEIL3 was related to treatment sensitivity. In the present research, we found that NEIL3 affected cell cycle activity by regulating the ATR/CHK1 pathway and ultimately promoting the sensitivity of PCa cells to radiotherapy.

Radiotherapy is one of the main methods of PCa treatment, and is supposed to cause DNA base breaks, DNA single strand breaks (SSBs), and DNA double-strand breaks (DSBs)^30^. Tumor cells often demonstrated G2 arrest after radiotherapy^31,32^. The TOPBP1/ATR/CHK1 pathway is regarded as an important checkpoint in G2 phase arrest and is considered to be regulated by many factors^28,29^. NEIL3 shares high homology with the apurinic/apyrimidinic endonuclease 2 (APE2) Zf-GRF domain, which can activate the ATR-CHK1 DNA damage response (DDR) pathway^33^. In addition, another study demonstrated that loss of NEIL3 enhances sensitivity to ATR inhibitors in glioblastoma cells^34^. In summary, NEIL3 may affect the activation of the ATR/CHK1 pathway in many different ways, which requires further exploration.

Additionally, the function of NEIL3 has been reported to be more complicated and not fully elucidated^17–19^. It has been reported that somatic mutation burden exhibits significant inverse correlations with NEIL1 and NEIL2 expression levels but a significant positive correlation with NEIL3 expression levels^35^. A high tumor mutation burden will produce many new antigens, which in turn activate more tumor-specific T cells and eventually enhance the sensitivity of immunotherapy. In addition, some studies have shown that deficiency in NEIL3 is associated with increased lymphocyte apoptosis^36^. Therefore, the loss of NEIL3 may also be an important reason why PCa patients are not sensitive to existing anti-PD-1 treatment.

A limitation of this study is that we only explored the role of NEIL3 in the radiosensitivity of PCa. It is well known that CRPC and NEPC are resistant not only to radiotherapy but also to ADT, chemotherapy, and even immunotherapy. Although some studies have shown that NEIL3 may play a role in treatment resistance, future studies should focus on evaluating the role of NEIL3 in other treatments.

## Conclusions

It is our novel discovery that some genes may have no effect on tumor progression or metastasis, but their existence is a prerequisite to ensure sensitivity to current treatment. Here, we found that loss of NEIL3 activated radiotherapy resistance in the progression of prostate cancer potentially *via* the ATR/CHK1 pathway. NEIL3 may serve as a diagnostic or therapeutic target for CRPC or NEPC patients.
