# Supplementary material for: Loss of NEIL3 activates radiotherapy resistance in the progression of prostate cancer
Source: Cancer Biol Med. 2021 Oct 1;19(8):1193–210. doi: 10.20892/j.issn.2095-3941.2020.0550 (PMC9425180; doi:10.20892/j.issn.2095-3941.2020.0550)
Supplement: Supplementary file 1 [file cbm-19-1193-s001.pdf]

Supplementary materials

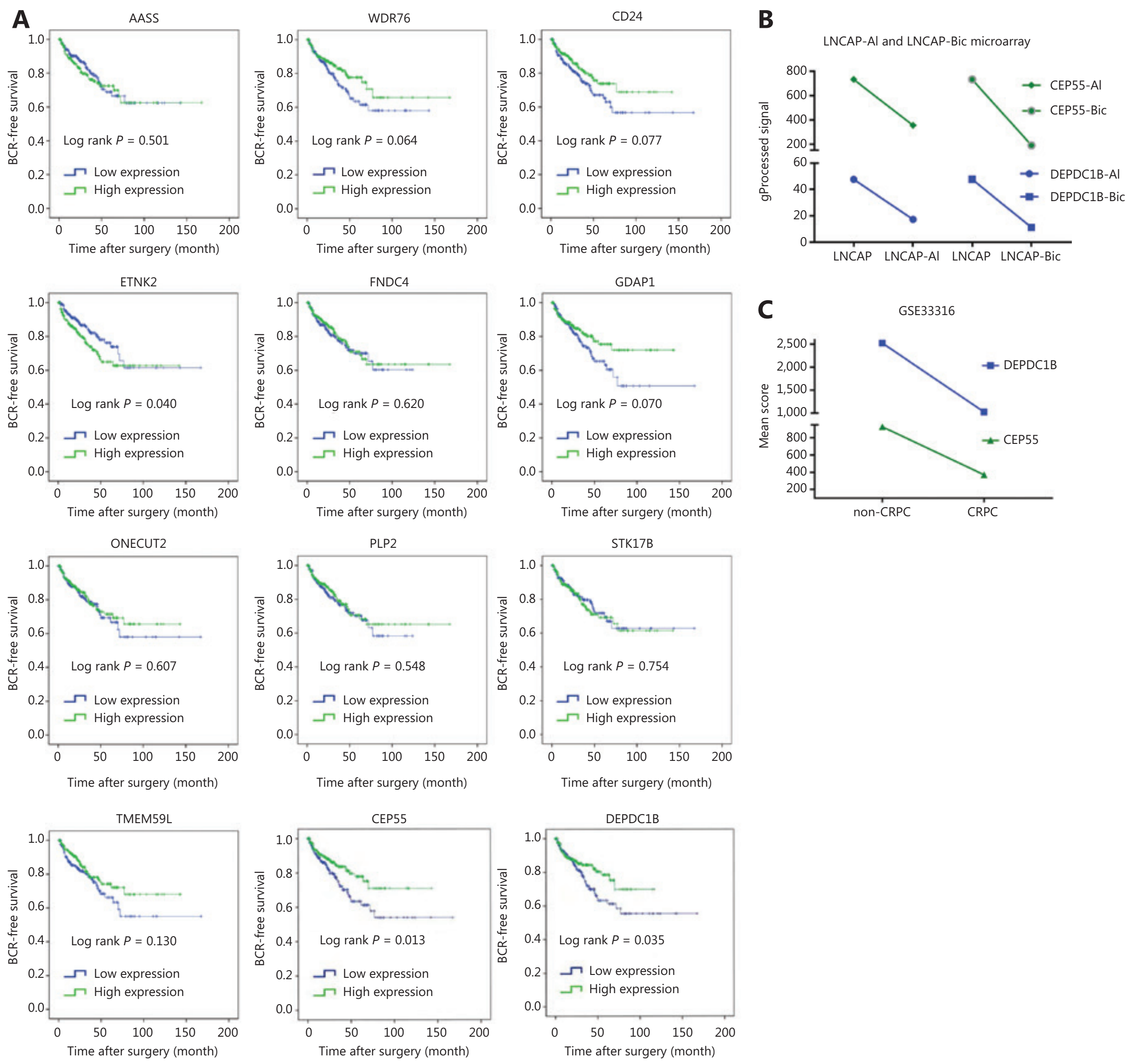

Figure S1 TCGA and database analysis. (A) TCGA BCR survival analysis of selected AASS, CD24, ENTN2, FNDC4, GDAP1, ONECUT2, PLP2, STK17B, TMEM59L, WDR76 CEP55 and DEPDC1B. (B, C) The expression of CEP55 and DEPDC1B in our CRPC microarray and GSE33316.

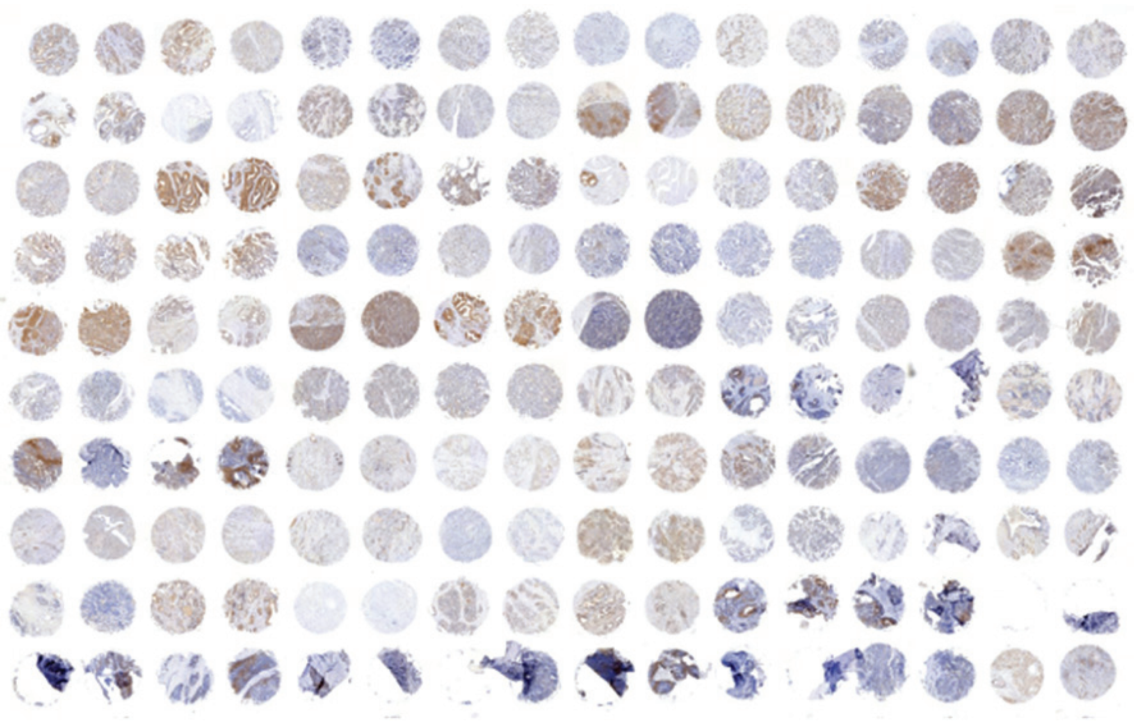

Figure S2 Photomicrographs of the immunohistochemical staining of NEIL3 in the TMA (160 PCa patients).

A

	Gene name	Sense (5′–3′)	Antisense (3′–5′)
siRNA1	NE IL3-Homo-829	GCA GGACUUGCUCUCUCUATT	UAGAGAGAGCAAGUCCUGCTT
siRNA2	NE IL3-Homo-1556	GCAACCCUGGAUAUU CUAATT	UUAGAAUAUCCAGGGUUGCTT

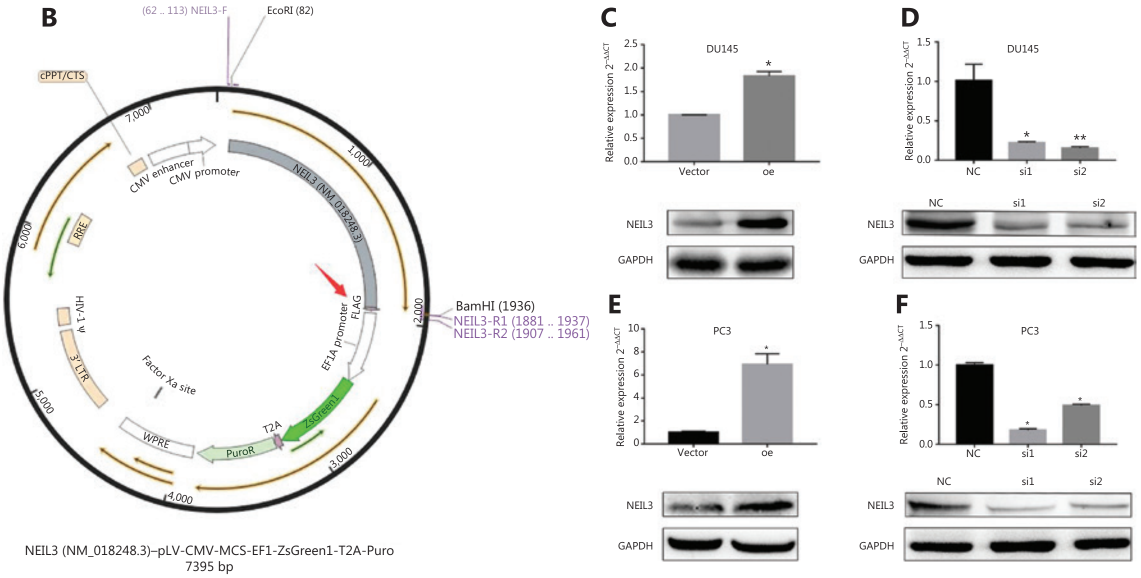

Figure S3 Construction of the NEIL3 overexpression and downregulation of the PCa cell line. (A) The sequences of siRNA used in this study. (B) The NEIL3 overexpression plasmid map. (C–F) qRT-PCR and Western blot analysis of NEIL3 expression levels in NEIL3-overexpressing cells (C, E), NEIL3-knockdown cells (D, F), and control cells; **P* < 0.05 and ***P* < 0.01, the versus vector or NC group.

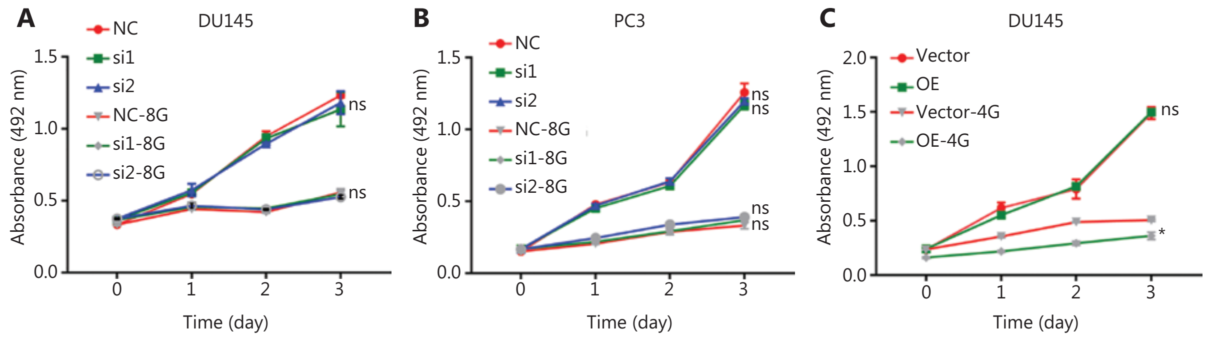

Figure S4 8 Gray dose caused the death of most PCa cells, and DU145 cells were more sensitive to radiotherapy. (A, B) The MTS assay was used to test cell viability in the DU145 (A) and PC3 (B) cell lines of the NEIL3 knockdown and control groups before and after radiotherapy using an 8 Gray dose. (C) The MTS assay was used to test cell viability in the DU145 cell line of the NEIL3 overexpression and control groups before and after radiotherapy using a 2 Gray dose; *P < 0.05, versus the corresponding vector group.

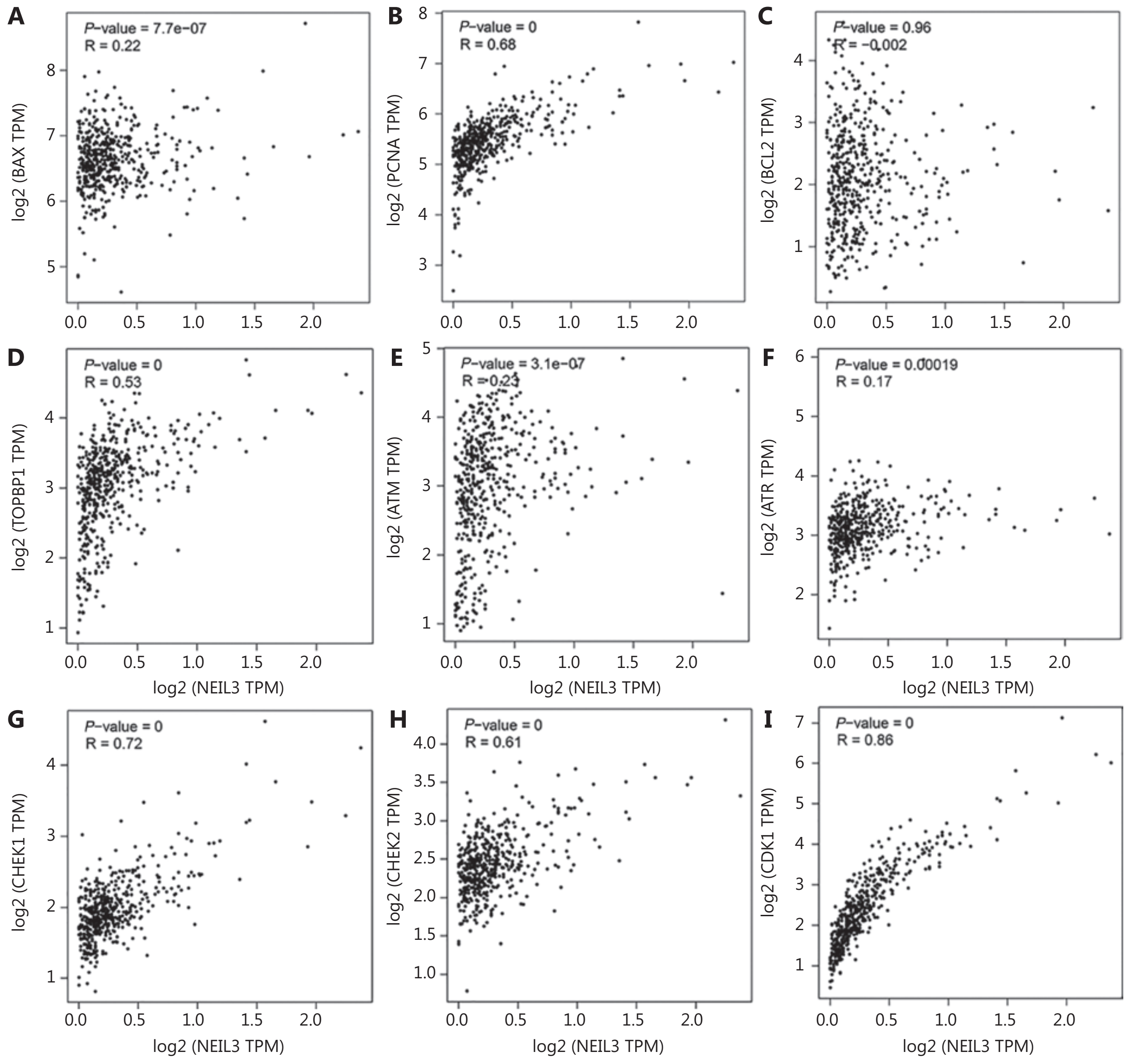

Figure S5 The correlation between NEIL3 and apoptosis, cell cycle related marker. (A–C) The correlation between NEIL3 and apoptosis related marker: BAX, PCNA and BCL2. (D–I) The correlation between NEIL3 and cell cycle related marker: TOPBP1, ATM, ATR, CHK1, CHK2 and CDK1.

Table S1 Correlation of NEIL3 expression with clinico-pathologic characteristics of PCa patients in TCGA database (all patients)

Clinical features	Total patients, *n*	Low, *n* (%)	High, *n* (%)	*P*
Age, years				0.056
≤ 65	354	187 (52.8%)	167(47.2%)	
> 65	143	62 (43.4%)	81 (56.6%)	
Gleason score				0.000***
≤ 7	292	184 (63%)	108 (37%)	
> 7	205	65 (31.7%)	140 (68.3%)	
Serum PSA levels, ng/mL				0.059
≤ 4	415	216 (52%)	199 (48%)	
> 4	27	9 (33.3%)	18 (66.6%)	
T stage				0.000***
T1-T2	187	122 (65.2%)	65 (34.8%)	
T3-T4	303	121 (39.9%)	182 (60.1%)	
Lymph node metastasis				0.006**
N0	346	173 (50%)	173 (50%)	
N1	79	26 (32.9%)	53 (67.1%)	
Distant metastasis				1.000
M0	454	225 (49.6%)	229 (50.4%)	
M1	3	1 (33.3%)	2 (66.6%)	

Cut off value of NEIL3 score: medium; PSA, prostate-specific antigen; T, tumor; N, node; M, metastasis. ***P* < 0.01; ****P* < 0.001.

Table S2 Correlation of NEIL3 expression with clinico-pathologic characteristics of PCa patients in TMA (160 patients)

Clinical features	Total patients, *n*	Low, *n* (%)	High, *n* (%)	*P*
Age, years				0.094
≤ 65	50	14 (28%)	36 (72%)	
> 65	110	46 (41.8%)	64 (58.2%)	
Gleason score				0.015*
≤ 7 (3 + 4)	46	24 (52.2%)	22 (47.8%)	
≥ 7 (4 + 3)	114	36 (31.6%)	78 (68.4%)	
T stage				0.302
T1-T2	72	26 (36.1%)	46 (63.9%)	
T3-T4	46	21 (45.7%)	25 (54.3%)	
Lymph node metastasis				1.000
N0	112	45 (40.2%)	67 (59.8%)	
N1	6	2 (33.3%)	4 (69.7%)	

Cut off value of Immuno-reactivity score: 6 (> 6, High; ≤ 6, Low.); T, tumor; N, node; M, metastasis.**P* < 0.05.

Table S3 The sequences of primers used in this study

Gene name	Forward (5′–3′)	Reverse (5′–3′)
NEIL3	GCAGTAAACACAACCGCCTC	AAGGACAAATCTGCCCATT CAA
GAPDH	CAAGGCTGAGAACGGGAAG	TGAAGACGCCAGTGGACTC
